# EWAS: epigenome-wide association studies software 1.0 – identifying the association between combinations of methylation levels and diseases

**DOI:** 10.1038/srep37951

**Published:** 2016-11-28

**Authors:** Jing Xu, Di Liu, Linna Zhao, Ying Li, Zhaoyang Wang, Yang Chen, Changgui Lei, Lin Gao, Fanwu Kong, Lijun Yuan, Yongshuai Jiang

**Affiliations:** 1 College of Bioinformatics Science and Technology, Harbin Medical University, Harbin, 150086, China; 2 Training Center for Students Innovation and Entrepreneurship Education of Harbin Medical University, Harbin, China; 3 Department of Ophthalmology, the Second Affiliated Hospital, Harbin Medical University, Harbin, China; 4 Department of Nephrology, The Second Affiliated Hospital, Harbin Medical University, Harbin, China

## Abstract

Similar to the SNP (single nucleotide polymorphism) data, there is non-random association of the DNA methylation level (we call it methylation disequilibrium, MD) between neighboring methylation loci. For the case-control study of complex diseases, it is important to identify the association between methylation levels combination types (we call it methylecomtype) and diseases/phenotypes. We extended the classical framework of SNP haplotype-based association study in population genetics to DNA methylation level data, and developed a software EWAS to identify the disease-related methylecomtypes. EWAS can provide the following basic functions: (1) calculating the DNA methylation disequilibrium coefficient between two CpG loci; (2) identifying the MD blocks across the whole genome; (3) carrying out case-control association study of methylecomtypes and identifying the disease-related methylecomtypes. For a DNA methylation level data set including 689 samples (354 cases and 335 controls) and 473864 CpG loci, it takes only about 25 min to complete the full scan. EWAS v1.0 can rapidly identify the association between combinations of methylation levels (methylecomtypes) and diseases. EWAS v1.0 is freely available at: http://www.ewas.org.cn or http://www.bioapp.org/ewas.

DNA methylation is an important epigenetic modification by adding a methyl group to the 5 position of cytosine, and forming 5-methylcytosine (5-mC)^1^,^2^. The DNA methylation associates with a number of key processes, such as the regulation of gene expression^3^,^4^, aging^5^,^6^, genomic imprinting^7^,^8^ and development^9^,^10^,^11^.

Recently years, with the development of chip and sequencing technology, such as Illumina Infinium HumanMethylation450 BeadChip (450 K methylation array) and bisulfite sequencing technology, it is possible for us to measure DNA methylation on a genome wide scale. Only for the GPL13534 platform 450k platform, including 485,577 probes) in NCBI GEO database^12^,^13^, at present, there are about 648 series including more than 42789 samples, and the number of samples is increasing rapidly. Under this background, the epigenome-wide association studies are developed. The epigenome-wide association studies aim to systematically identify epigenetic variants associated with complex diseases or phenotypes for case-control studies^14^,^15^. So far, many complex diseases have been successfully analyzed by using the epigenome-wide association studies, such as Parkinson’s disease^15^, Schizophrenia^16^, Psoriasis^17^.

Similar to linkage disequilibrium (LD) of single nucleotide polymorphisms (SNP), there is non-random association of the DNA methylation level between neighboring methylation loci. Here, we call the non-random association methylation disequilibrium (MD). Some previous studies also indicated that the decay distance of MD is about 1 kb ~10 kb^18^,^19^. For the case-control study of SNP data, a classical analysis is to identify complex disease-related haplotype (the combination of SNP alleles). The most popular software that can identify the disease-related haplotype is Haploview^20^. Here we extended the classic framework of haplotype-based association study in Haploview to DNA methylation data. Our aim is to develop a software EWAS that can identify the association between combinations of methylation levels (here, the combinations of methylation levels are called, methylecomtype) and diseases in case-control study. EWAS can be used to calculate the MD coefficient between any two DNA methylation loci, identify the MD blocks and perform the methylecomtypes-based case-control association study. We hope the classic framework of haplotype-based association study can be widely used in the epigenome-wide association studies.

## Methods

### DNA methylation level *β* value

The data used in the EWAS software is the methylation status *β*-values. *β*-value is usually defined as *β* = methylated signals/(methylated signals + unmethylated signals + 100). The *β*-values range from 0 to 1. The users can obtain the DNA methylation profile through the methylation chip (such as Illumina 450 K array) or the methylation sequencing (such as bisulfite sequencing).

### The DNA methylation disequilibrium (MD)

The DNA methylation disequilibrium is derived from the concept of SNP linkage disequilibrium (LD)^21^,^22^,^23^,^24^. The LD means that the alleles of two SNPs were non-random association. Here, we changed the DNA methylation *β* value into two levels (H: high DNA methylation level and L: low DNA methylation level) based on some threshold T (e.g. T = 0.5), and defined the MD and related concepts as following:

(1) **methylecomtype (methylation levels combination types):** we defined the methylecomtype as the combinations of methylation levels (H or L). For example, we supposed that there are three CpG loci, and each locus had two levels: H and L. For all the samples, there may be total 2^3^ = 8 types of methylecomtypes: HHH, HHL, HLH, HLL, LHH, LHL, LLH and LLL. Each sample is one of the eight types of methylecomtypes. If there is an association between loci, the types of methylecomtypes will less than eight.

(2) **methylation equilibrium (ME):** We supposed that there were two methylation loci M1 and M2. M1 had two levels H1 and L1, and M2 had two levels H2 and L2. If there is no association between the methylation levels of two loci M1 and M2, we believed that M1 and M2 were Methylation equilibrium (ME). In other words, the methylation level of M1 locus is independent of the methylation level of M2 locus. We then described the ME by using the principle of independence:


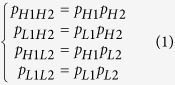


where *p*_*H*1*H*2_ is the frequency of methylecomtype H1H2, *p*_*L*1*H*2_ is the frequency of methylecomtype L1H2, *p*_*H*1*L*2_ is the frequency of methylecomtype H1L2, *p*_*L*1*L*2_ is the frequency of methylecomtype L1L2, *p*_*H*1_ is the frequency of high DNA methylation level H1 at M1 locus, *p*_*L*1_ is the frequency of low DNA methylation level L1 at M1 locus, *p*_*H*2_ is the frequency of high DNA methylation level H2 at M2 locus, and *p*_*L*2_ is the frequency of low DNA methylation level L2 at M2 locus. These frequencies can be calculated as follows:


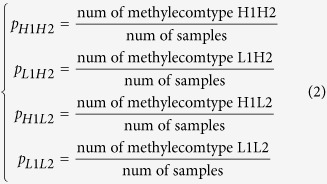



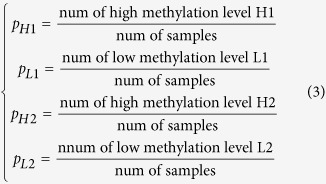


(3) **MD (methylation disequilibrium):** We still assumed that there are two methylation loci M1 and M2. M1 had two levels: H1 and L1, and M2 had two levels H2 and L2. If there is non-random association between the methylation levels of two loci M1 and M2, we believed that M1 and M2 were Methylation disequilibrium (MD). We then gave the general methylation disequilibrium coefficient *gd* as follows^25^,^26^:


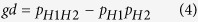


### Standardization of *gd*

In this study, we provided two approaches, which are commonly used in LD analyses (LD coefficient *D*′ and *r*^2^), to standardize *gd*. The standardized MD coefficient *gd*′ and *gr*^2^ can be calculated as^27^:


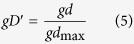



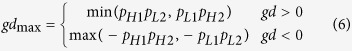


The range of *gD*′ is from 0 to 1^25^.

*gr*^2^ can be calculated as^28^:


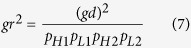


The range of *gr*^2^ is also from 0 to 1.

### MD block

In this study, we defined MD block as a chromosome region consisting of a group of closely related DNA methylation loci. That is, in a block region, DNA methylation loci were high MD with each other. Here, we used the Gabriel *et al*.’s algorithm to identify the MD blocks^29^.

### Methylecomtype based association study

For each methylecomtype in a MD block, we carry out a case/control *χ*^2^ test to identify whether the methylecomtype is associated with a disease or phenotype. At the same time, we also calculate OR ratio and its 95% CI for each methylecomtype in every block.

## Results

### Overview of the EWAS

EWAS is written entirely using Java, and can be run on any platform which has installed Java 1.8 or later version. By using command line, EWAS is able to process whole genome DNA methylation data with hundreds of samples in acceptable time. EWAS has three basic functions: (1) calculating the MD coefficient *gD*′ and *gr*^2^ between any two DNA methylation loci in a genome region; (2) scanning the entire genome using an elastic sliding window to identify the MD blocks; (3) identifying the disease-related methylecomtypes. Users can easily complete the epigenome wide gMaplotype association by executing the command:


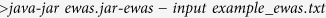


### The input data

For case/control association study, the input file should include the information of sample status and DNA methylation levels. The statuses of samples were listed in the first row of the input file. In EWAS, we use 1,0 or −1 to represent case, control or unknown status. From the second row, each line in the input file is a DNA methylation locus and its detailed information including: name of the locus, chromosome number, physical position in chromosome, and DNA methylation level *β*-values for each of the samples. To facilitate subsequent calculation, we firstly sort the DNA methylation loci based on their chromosome number and physical position. Then all the DNA methylation level *β*-values were changed into two levels (H: high DNA methylation level and L: low DNA methylation level). All the sorted data were saved in a temporary file ‘out_sort.txt’. Users also can use the temporary file to carry out their personalized analysis.

If users only want to calculate the MD coefficients, identify the MD blocks, and calculate the frequencies of methylecomtypes, they only need to set the statuses of all samples to −1. The example data (example_coefficient.txt and example_block.txt) can be found in EWAS website.

### Calculating the MD coefficient *gD*′ and *gr* ^2^

EWAS can calculate the MD coefficients *gD*′ and *gr*^2^ (see method) between different DNA methylation loci. The *gD*′ and *gr*^2^ are used to measure whether there is association between DNA methylation levels at two methylation loci. The ranges of both *gD*′ and *gr*^2^ are from 0 to 1. Larger *gD*′ or *gr*^2^ represents the stronger associations of DNA methylation levels between two loci. For a given list of DNA methylation data (see ‘The input data’ section or example file ‘example_coefficient.txt’), EWAS can output MD information for each pair of DNA methylation loci. The output results include: the name of pair-wise DNA methylation loci, the chromosome number, the position, the MD coefficients *gD*′, the MD coefficients *gr*^2^. Users can use the following command to calculate and output MD coefficients:


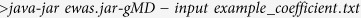


### Identifying MD blocks and calculating methylecomtype frequency

EWAS use the Gabriel *et al*.’s algorithm to identify the MD blocks^29^. For a chromosome region, we first calculated the MD coefficients *gD*′ for each pair-wise DNA methylation loci. If 95% of MD coefficients *gD*′ show strong MD, then this region is considered to be a MD block by EWAS. A part of Java codes and methods that identify the block can be found in Haploview^20^.

In this study, we use a sliding window to scan the entire genome. For the larger block, we design an elastic sliding window method to avoid breaking the real MD block structure. Firstly, we set a fixed length of sliding window (such as 20 loci) to identify the MD blocks in the window. If the last locus in the window is not located in a block, we will slide the window to the next locus. If the last locus in the window is located in an identified block, the block is likely to be broken by the border of the window. Then we increase the size of sliding window to two fold (such as 40 loci) size of fixed window. By using this strategy, we can scan the entire genome in a single run.

For each identified block, EWAS can also integrate the types of methylecomtype (methylation levels combination types) in the block region, and calculate the frequencies of methylecomtypes. For more details, see method.

For a given list of DNA methylation data (see ‘The input data’ section or example file ‘example_block.txt’), users can identify the MD blocks and calculated the frequency of methylecomtype using the following command:


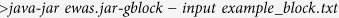


The output results include: the name of a block, the chromosome number, the start and end position, and the list of DNA methylation loci in the block, the methylecomtype in the block and the frequency of methylecomtype.

### Epigenome-wide association studies for methylecomtype

For a MD block region, EWAS will count the number of methylecomtype in case samples and control samples, and perform a chi-square test to identify the significant methylecomtypes associated with diseases or phenotypes. The association test is integrated into the framework of epigenome-wide methylecomtype association study.

Then we describe the complete process of epigenome-wide methylecomtype association study. There are five main steps: (1) sort the DNA methylation loci; (2) change DNA methylation *β*-values into two levels H and L; (3) scan the genome and identify the MD blocks; (4) calculate the frequency of methylecomtypes in MD block region; (5) carry out chi-square test to identify the significant methylecomtypes. Researchers can use the command ‘-ewas’ to automatically complete all the five steps. The output file includes the following information: the chromosome number, the name of a block, the start and end position, the list of DNA methylation loci in the block, the frequency of methylecomtype in case and control, the chi-square value, P-values for chi-square test, OR and its 95% CI.

### Running time

**Figure 1:**
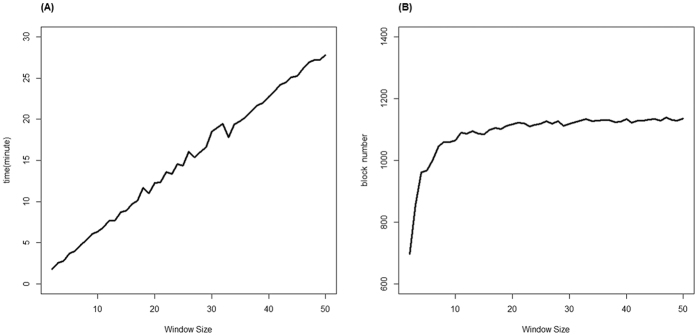
(**A**) The relationship between the window size and running time; (**B**) the relationship between the window sizes and block numbers.

We tested the running time using a EWAS dataset (GSE42861) downloaded from GEO database (https://www.ncbi.nlm.nih.gov/geo/query/acc.cgi?acc=GSE42861)^30^. The dataset includes 689 samples (354 Rheumatoid arthritis case and 335 normal controls). The DNA methylation levels of samples were tested by using Illumina 450 K array. There were total 473864 loci on the autosomal chromosomes, for each loci, the *β*-value was used to present the methylation status. All the tests were run on a personal computer with 2.5 GHz Intel(R) Core(TM) i3-3120M CPU and 4 G of RAM. We firstly sorted the data based on their chromosome physical location. This process takes about 12 minutes. Then we used the sorted data to carry out Epigenome-wide methylecomtype association studies (identify the MD blocks, calculate the frequency of methylecomtypes, and carry out chi-square test) for different window sizes. The range of window size is from 2 loci to 50 loci. From Fig. 1A we can see that the running time increased with the window size increased. Figure 1B showed the relationship between the window sizes and block numbers. We observed that the number of blocks reached a stable state and was almost not changed when the window size is larger than 20. Therefore, the default window size in EWAS software was set to 20. This process took about 13 minutes. This indicated that the EWAS software can process whole epigenome scale datasets in a feasible time on personal computers (PC).

## Discussions

In this study, we developed a DNA methylation methylecomtype analysis tools EWAS v1.0. We extended the classic framework of SNP haplotype-based association study to DNA methylation methylecomtype-based association study. EWAS v1.0 can not only calculate the DNA methylation disequilibrium coefficient between two CpG loci, but also calculate the MD between methylation loci and identify the disease-related methylecomtypes.

In addition, EWAS v1.0 also can carry out epigenome-wide T test for beta-value of single DNA methylation locus. For a given list of DNA methylation data (see ‘The input data’ section or example file ‘example_ewas.txt’), users can calculate the T test value and p value for each methylation locus using the following command:


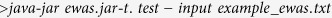


Due to technical limitations, we only consider the methylecomtype (methylation levels combination types). We change the DNA methylation *β*-values into two levels (H: high DNA methylation level and L: low DNA methylation level) based on some threshold T (e.g. T = 0.5) for analyzing the association of the DNA methylation level between neighboring methylation loci, but there is some useful information losing in this process. With the development of technology, the detection of methylaltion loci will be more accurate. We can develop some meplotype analysis modules for the further study of complex diseases.

### Future

We will develop our software in the future from the following aspects:

1. epigenome-wide association study for SMP (single methylation polymorphism);
2. epigenome-wide association study for meplotype (methylation haplotype);
3. epigenome-wide association study for gene region;
4. epigenome-wide association study for KEGG pathway;
5. epigenome-wide association study for GO categories;
6. epigenome-wide association study for network;
7. epigenome-wide association study for interacting with genetic marker;
8. epigenome-wide association study for gene expresssion;

The data resource and software will be found on the EWAS (epigenome-wide association study) website: http://www.ewas.org.cn. or http://www.bioapp.org/ewas.

## Additional Information

**How to cite this article**: Xu, J. *et al*. EWAS: epigenome-wide association studies software 1.0 – identifying the association between combinations of methylation levels and diseases. *Sci. Rep.* **6**, 37951; doi: 10.1038/srep37951 (2016).

**Publisher's note:** Springer Nature remains neutral with regard to jurisdictional claims in published maps and institutional affiliations.
